# Circulating and extracellular vesicle-associated non-coding RNAs in diabetic retinopathy: a stage-aware framework for network-informed biomarker panels

**DOI:** 10.3389/fendo.2026.1970838

**Published:** 2026-10-21

**Authors:** Ji-Won Yu, Myung-Ji Choi, Dong Cheol Lee, Jae-Hyung Park

**Affiliations:** 1 Medical Course, School of Medicine, Keimyung University, Daegu, Republic of Korea; 2 Department of Ophthalmology, Keimyung University School of Medicine, Daegu, Republic of Korea; 3 Department of Physiology, Keimyung University School of Medicine, Daegu, Republic of Korea

**Keywords:** circulating biomarkers, diabetic retinopathy, extracellular vesicles, liquid biopsy, microRNA, non-coding RNA, risk stratification

## Abstract

Diabetic retinopathy (DR) is a progressive microvascular complication of diabetes in which prognosis and management change substantially across non-proliferative and proliferative stages. Current imaging accurately detects established structural and vascular abnormalities but provides limited insight into early molecular changes or the risk of clinically important transitions within non-proliferative diabetic retinopathy (NPDR). Circulating non-coding RNAs (ncRNAs), including microRNAs (miRNAs), long non-coding RNAs (lncRNAs), and circular RNAs (circRNAs), have emerged as candidate biomarkers because they regulate pathways involved in endothelial dysfunction, inflammation, oxidative stress, and angiogenesis and can be detected in blood and extracellular vesicle (EV)-associated fractions. In this structured narrative review, we synthesize circulating and EV-associated ncRNA evidence within a stage-aware framework aligned with three clinical use cases: early screening, risk stratification within NPDR, and monitoring at the severe NPDR–early proliferative diabetic retinopathy boundary. miRNAs currently provide the most mature evidence for stage-associated expression patterns and, in selected studies, stage-discriminatory performance. By contrast, circulating lncRNA and circRNA evidence remains limited and is predominantly cross-sectional and advanced-stage associated, supporting their use mainly as complementary, network-informing components. EV association may improve RNA stability and enrich disease-relevant signals, but it does not establish retinal origin, and differences in biofluid source, EV isolation and characterization, normalization, and systemic confounders limit cross-study comparability. Importantly, direct evidence for clinically relevant adjacent-stage discrimination and longitudinal prediction remains scarce. We therefore propose that future biomarker development should prioritize stage-aware, network-informed multi-ncRNA panels, standardized pre-analytical and EV workflows, adjustment for major clinical confounders, and longitudinal imaging-integrated validation to determine whether these biomarkers add value beyond conventional retinal staging.

## 1 Introduction

### 1.1 Diabetic retinopathy as a progressive, stage-dependent disease

Diabetic retinopathy (DR) is one of the most common microvascular complications of diabetes mellitus and remains a leading cause of vision loss worldwide (1). Importantly, DR is a progressive, stage-dependent disease that evolves from non-proliferative diabetic retinopathy (NPDR) to proliferative diabetic retinopathy (PDR). The latter is characterized by pathological angiogenesis and vision-threatening complications (2). This progression reflects cumulative molecular and cellular alterations, including endothelial dysfunction, pericyte loss, chronic inflammation, and ischemia-driven neovascular responses (2, 3). As clinical outcomes and therapeutic strategies differ substantially across disease stages, a better understanding of stage-specific disease mechanisms is essential for effective diagnosis and management (4).

### 1.2 Limitations of current diagnostic and monitoring strategies

Current diagnostic approaches for DR primarily rely on imaging modalities, such as fundus photography and optical coherence tomography, which identify structural or vascular abnormalities after significant retinal damage has occurred (5). While these techniques are effective for detecting established disease and guiding treatment decisions, they have limited sensitivity for early molecular changes and provide restricted insight into the dynamic progression of the disease (6). Concurrently, circulating protein biomarkers, including vascular endothelial growth factor (VEGF), have been extensively studied, particularly in therapeutic contexts; however, their diagnostic performance is hindered by poor specificity and inadequate discrimination between disease stages (6, 7). As a result, there is a critical unmet need for minimally invasive biomarkers that can facilitate early detection and longitudinal monitoring of DR progression.

### 1.3 Rationale for non-coding RNAs and extracellular vesicles

Non-coding RNAs (ncRNAs), including microRNAs, long non-coding RNAs, and circular RNAs, have emerged as key regulators of gene networks involved in angiogenesis, inflammation, oxidative stress, and cell survival in diabetic retinopathy (6, 8). Experimental evidence suggests that these ncRNAs act as upstream modulators of major pathogenic pathways, including VEGF-related signaling, across multiple retinal cell types (9). Notably, ncRNAs can be stably detected in circulation, particularly when encapsulated within extracellular vesicles (EVs), which protect them from degradation and facilitate intercellular communication (10). These features position EV-associated ncRNAs as promising candidates for circulating biomarkers that may reflect underlying retinal pathology (11).

### 1.4 Scope and differentiation of this review

Previous reviews have addressed the pathogenic and therapeutic roles of ncRNAs in diabetic retinopathy (9), while recent systematic reviews have increasingly focused on circulating or EV-associated ncRNAs as potential biomarkers (11–13). For example, Upadhayay et al. systematically reviewed serum- and plasma-derived circulating miRNAs in type 2 diabetic retinopathy, focusing on their diagnostic potential and pathogenic relevance (13). In addition, a recent diagnostic-accuracy systematic review and meta-analysis evaluated circulating miRNAs across broad clinical comparisons, including diabetes without DR versus DR and NPDR versus PDR (12).

The present review addresses a complementary but distinct question. Rather than primarily assessing whether individual circulating miRNAs can distinguish broad disease categories, we organize circulating and EV-associated ncRNAs within a clinically oriented, stage-aware framework aligned with specific decision points across the DR continuum: early screening, risk stratification within NPDR, and progression monitoring toward PDR. We integrate miRNAs, lncRNAs, and circRNAs and interpret them within EV-associated and competing endogenous RNA network contexts (11), while distinguishing broad stage-associated signals from evidence that directly supports stage discrimination or prediction. Particular emphasis is placed on clinically relevant adjacent-stage transitions, including moderate versus severe NPDR and severe NPDR versus early PDR, for which current evidence remains limited (12). This framework is intended to move beyond a catalog of individual biomarkers and to identify the methodological and validation requirements needed for stage-aware ncRNA panels to complement imaging-based assessment in clinical practice.

This review is based on a structured narrative synthesis rather than a formal systematic review or meta-analysis. Literature searches were conducted in PubMed, Scopus, and Web of Science using predefined combinations of the following terms: “diabetic retinopathy”, “non-coding RNA”, “microRNA” or “miRNA”, “long non-coding RNA” or “lncRNA”, “circular RNA” or “circRNA”, “extracellular vesicle” or “exosome”, “biomarker”, and “circulating”. Search terms were combined using Boolean operators (AND/OR) and adapted as appropriate for the syntax of each database. Searches were limited to English-language articles published through June 2026, with the final search performed on June 30, 2026. Reference lists of retrieved articles and relevant review papers were additionally screened to identify potentially relevant studies not captured by the primary database search. Retrieved records were first screened based on titles and abstracts, followed by full-text assessment of potentially relevant articles. Studies were included in the stage-resolved biomarker synthesis when they met the following criteria: (i) human studies reporting circulating or EV-associated ncRNA measurements in subjects with diabetic retinopathy; (ii) explicit clinical classification of DR status or severity; and (iii) availability of interpretable expression directionality relative to a defined comparator group. Studies limited to tissue-level or *in vitro* ncRNA expression without circulating biomarker data, studies lacking clinically interpretable DR staging, and reports without sufficient information on ncRNA directionality were excluded from the stage-resolved synthesis. Mechanistic experimental studies were retained when relevant to support the biological interpretation of candidate ncRNAs and their associated regulatory pathways. Given the structured narrative scope of this review, no formal risk-of-bias assessment or meta-analytic pooling was performed. Instead, evidence strength was qualitatively appraised using the review-specific evidence-grading framework described in Section 5.5 and **Tables 1**–**3**.

**Table 1:** Stage-resolved miRNA evidence summary and candidate panels in diabetic retinopathy.

miRNA	Stage association/proposed clinical role	Direction of change	Diagnostic performance /effect size	N/validation status	No. of studies/evidence level	Ref.
miR-3976	Screening (early DR)	↑	log2FC 4.04 (DR vs DM), p < 0.001; AUC NR	n=18/discovery + internal qPCR validation	1 · Low	(46)
miR-431-5p	Advanced DR/PDR-stage EV biomarker	↑	FC ×2 in PDR vs healthy/DM/non-PDR	n=160/Discovery + qRT-PCR validation	1 · Low	(61)
miR-23a-3p	Screening (DR vs no DR)	↓	p = 0.013; AUC NR	n=45/single cohort; no independent validation	1 · Low	(47)
miR-15a-5p	DR-associated; proposed risk stratification	↓	p = 0.027; AUC NR			
miR-126-5p	Screening (early/mild NPDR)	↓	qRT-PCR down-regulation; AUC NR	RNA-seq n=6; qRT-PCR validation n=15; clinical cohort n=86	1 · Low	(48)
miR-26a-5p	Screening (early DR; RNFL)	↓	Pearson r with superior-quadrant RNFL; AUC NR			
miR-21	Severity-associated; NPDR–PDR discrimination	↑	AUC 0.89 (95% CI 0.86–0.96), Se 82–86%, Sp 84–95% (PDR vs NPDR) (60); AUC 0.825 (95% CI 0.778–0.872), Se 66.1%, Sp 90.4% (DR); AUC 0.830 (0.761–0.900), Se 72.5%, Sp 79.5% (PDR) (51)	n=200/239/80/multistage validation in one study; 3 independent cohorts	3 · Moderate	(51, 52, 60)
miR-25-3p	Severity-associated (DR continuum)	↑	3-miRNA panel AUROC 0.931 (95% CI 0.853–1.000), Se 85%, Sp 85% (DR detection)	n=40/internal validation; no external cohort	1 · Low	(53)
miR-320b		↑				
miR-495-3p		↓				
miR-16-5p	DR-associated; proposed screening	↑	FC ≥ |8.0| (pooled screening only; AUC NR)	n=30 (pooled discovery)/discovery only; not individually validated	1 · Low	(53)
miR-155	Severity-associated (↑ with stage)	↑	Gradient PDR > BDR > NDR > NC; AUC NR	n=78/single cohort; no independent validation	1 · Low	(68)
miR-93	Severity-associated (spectrum)	↓	AUC 0.99 (95% CI 0.97–1.00), Se 97%, Sp 95% (NPDR vs DM); AUC 0.79 (95% CI 0.69–0.88), Se 85%, Sp 63% (NPDR vs PDR)	n=240/single cohort; no independent validation	1 · Low	(49)
miR-152	Severity-associated (spectrum)	↑	AUC 0.97 (95% CI 0.91–1.00), Se 93%, Sp 91% (PDR vs DM); AUC 0.88 (95% CI 0.81–0.96), Se 85%, Sp 80% (NPDR vs PDR)			

Evidence level was qualitatively assigned based on independent replication, directionality consistency, stage resolution, and validation or longitudinal support: low, single or unreplicated studies with limited stage resolution; moderate, replicated and/or independently validated findings with generally consistent directionality but limited adjacent-stage or longitudinal evidence; and high, consistent findings across multiple independent cohorts with clinically relevant stage discrimination and robust validation. This review-specific framework is not a formal GRADE assessment. Stage-associated indicates cross-sectional stage or severity associations; stage-discriminatory indicates direct evaluation between predefined clinical stage groups; and predictive indicates longitudinal association with subsequent progression or clinical outcomes. Diagnostic performance metrics are reported as published, including 95% CIs, sensitivity, and specificity where available; NR indicates not reported. Panel-derived metrics are explicitly distinguished from individual-miRNA performance and are not directly comparable across studies. FC, fold change; AUC, area under the curve; AUROC, area under the receiver operating characteristic curve; Se, sensitivity; Sp, specificity; CI, confidence interval; ↑, increased; ↓, decreased.

**Table 2:** Stage-associated circulating and EV-associated lncRNAs in diabetic retinopathy: evidence strength and study design.

lncRNA	Stage relevance	Direction of change	Number of studies	Evidence level	Study design	Ref.
PPT2-EGFL8 (EV-associated)	Non-DR → NPDR → PDR	↓ in PDR	1	Low	Cross-sectional (EV profiling)	(69)
NR2F1-AS1	Healthy → T2DM → NPDR → PDR	↑ with increasing DR severity	1	Low	Cross-sectional (stage-stratified)	(70)
MIAT	NPDR → PDR	↑ in PDR	1	Low	Cross-sectional	(71)
HOTTIP	NPDR → PDR	↑ in PDR	1	Low	Cross-sectional	(71)
SNHG16	NPDR → PDR	↑ in PDR	1	Low	Cross-sectional	(71)

Evidence level was qualitatively assigned using the review-specific framework described in Section 5.5, considering the number of independent studies, directionality agreement, stage specificity and granularity, and the availability of independent or longitudinal validation. Low evidence generally indicates findings supported by a single primary study without independent replication, whereas moderate evidence requires support from multiple independent studies and/or validation with generally consistent directionality. Because most circulating lncRNA candidates in diabetic retinopathy are currently supported by single cross-sectional studies, they should be interpreted as advanced-stage-associated or context-defining biomarkers rather than validated standalone stage-discriminative markers. EV, extracellular vesicle; NPDR, non-proliferative diabetic retinopathy; PDR, proliferative diabetic retinopathy; ↑, increased; ↓, decreased.

**Table 3:** Stage-associated circulating and EV-associated circRNAs in diabetic retinopathy: evidence strength and study design.

circRNA	Stage relevance	Direction of change	Number of studies	Evidence level	Study design	Ref.
hsa_circ_0001953	Healthy → NPDR → PDR	↑ in PDR	1	Low	Cross-sectional (diagnostic validation)	(72)
circMKLN1 (EV-associated)	Control → DM → NPDR → PDR	↑ with increasing DR severity	1	Low	Cross-sectional (EV sequencing)	(73)
circFndc3b † (hsa_circ_0006156)	Control → PDR †	↑ in PDR	1	Low	Cross-sectional (EV-associated validation)	(74)

Evidence level was qualitatively assigned using the review-specific framework described in Section 5.5, considering the number of independent studies, directionality agreement, stage specificity and granularity, and the availability of independent or longitudinal validation. Low evidence generally indicates findings supported by a single primary study without independent replication, whereas moderate evidence requires support from multiple independent studies and/or validation with generally consistent directionality. Because current circulating circRNA evidence in diabetic retinopathy is based largely on small, cross-sectional studies with limited NPDR sub-stage resolution and no independent replication, these candidates should be interpreted as exploratory advanced-stage-associated biomarkers rather than validated standalone stage-discriminative markers. EV, extracellular vesicle; NPDR, non-proliferative diabetic retinopathy; PDR, proliferative diabetic retinopathy. ^†^circFndc3b was evaluated in a PDR-versus-cataract control comparison without NPDR sub-stage data, representing the lowest stage resolution among the three circRNA candidates listed; ↑, increased.

## 2 Existing blood-based biomarkers for diabetic retinopathy and their limitations

### 2.1 Protein biomarkers and anti-VEGF therapy

A wide range of circulating protein biomarkers has been investigated in diabetic retinopathy, with vascular endothelial growth factor (VEGF) receiving particular attention due to its central role in pathological angiogenesis (2, 7). Elevated VEGF levels and other angiogenic or inflammatory mediators have been reported, especially in patients with proliferative diabetic retinopathy, and these levels correlate with retinal neovascularization and disease severity (14, 15). This biological relevance has led to the widespread clinical use of anti-VEGF agents as first-line therapy for advanced DR and diabetic macular edema (16, 17). However, circulating VEGF levels exhibit substantial inter-individual variability and are influenced by systemic hypoxia, inflammation, and comorbid conditions (14, 18). Importantly, therapeutic relevance does not necessarily translate into diagnostic utility, as anti-VEGF interventions are typically initiated after the disease becomes clinically apparent and do not aid in early detection or stage-specific diagnosis (6). As a result, circulating VEGF is poorly suited as a decision-support biomarker for stage-aware management, such as triaging patients for expedited imaging, tailoring surveillance intensity within NPDR, or triggering earlier referrals based on near-term progression risk.

### 2.2 Inflammatory and vascular biomarkers

In addition to VEGF, inflammatory and vascular biomarkers such as C-reactive protein, interleukins, tumor necrosis factor-α, intercellular adhesion molecule-1, and vascular cell adhesion molecule-1 have been linked to the presence and severity of diabetic retinopathy (19, 20). These markers reflect systemic inflammation and endothelial dysfunction, processes that contribute to DR pathogenesis (18). However, similar alterations are observed across various diabetic and cardiovascular complications, which limits their disease specificity and ability to reliably distinguish between NPDR and PDR (6, 20).

### 2.3 Metabolic and nutritional biomarkers

Metabolic and nutritional biomarkers, including lipid-related parameters, markers of oxidative stress, and vitamin D levels, have been investigated as potential blood-based indicators of DR risk (21, 22). Although several studies have reported associations between these markers and the prevalence or severity of DR, their clinical utility remains limited (21). These biomarkers are significantly influenced by lifestyle factors, glycemic control, medication use, and supplementation status, and thus serve primarily as systemic risk modifiers rather than direct indicators of retinal pathology (6, 23).

### 2.4 Unmet clinical needs

Collectively, existing blood-based biomarkers offer valuable insights into systemic disease associations but fail to meet key clinical needs in diabetic retinopathy (6, 12). Specifically, they do not enable early, minimally invasive screening, reliably discriminate between disease stages—particularly between NPDR and PDR—or support longitudinal tracking of disease progression. Furthermore, many of these markers reflect downstream consequences of established retinal damage rather than upstream molecular alterations that precede clinical manifestations (2). From a clinical decision-support perspective, these limitations are significant: current blood-based markers rarely assist in determining who should be prioritized for expedited imaging-based evaluation (triage), how surveillance intensity should be tailored within NPDR (routine vs. shortened follow-up), or when referral and escalation of care should be initiated to prevent the transition to severe NPDR or early PDR. Consequently, there is a need for minimally invasive biomarkers that complement imaging by providing stage-aware risk stratification and progression monitoring aligned with clinically actionable transition points. These limitations highlight the necessity for novel biomarker strategies that capture stage-dependent retinal pathophysiology, thereby providing a rationale for exploring non-coding RNA–based biomarkers in diabetic retinopathy (11).

## 3 Non-coding RNAs and extracellular vesicles in diabetic retinopathy

### 3.1 Overview of miRNAs, lncRNAs, and circRNAs

Non-coding RNAs (ncRNAs) are functional RNA molecules that do not encode proteins but play essential roles in regulating gene expression at both transcriptional and post-transcriptional levels (24). Among these, microRNAs (miRNAs) are short RNAs, approximately 18–25 nucleotides in length, that regulate gene expression by binding to complementary sequences in target messenger RNAs. This binding can lead to translational repression or mRNA degradation (25, 26). Long non-coding RNAs (lncRNAs), typically longer than 200 nucleotides, regulate gene expression through various mechanisms, including chromatin remodeling, transcriptional control, and post-transcriptional modulation (27, 28). Circular RNAs (circRNAs) are covalently closed RNA molecules generated by back-splicing events, characterized by high structural stability and resistance to exonuclease-mediated degradation (24, 29).

Increasing evidence from experimental and clinical studies indicates that these classes of ncRNAs are involved in key pathogenic processes of diabetic retinopathy, such as inflammation, oxidative stress, endothelial dysfunction, and pathological angiogenesis (8, 11). Importantly, several ncRNAs exhibit stage-associated expression patterns, with differential alterations observed between non-proliferative and proliferative diabetic retinopathy, underscoring their potential relevance for disease stratification and stage-specific assessment (12).

### 3.2 Extracellular vesicles as carriers of ncRNAs

Extracellular vesicles (EVs) are heterogeneous membrane-bound particles released by most cell types and serve as important mediators of intercellular communication (30). EVs can be categorized based on physical characteristics, biochemical composition, cellular origin, or biogenesis-related mechanisms. However, current nomenclature surrounding EVs advises caution in assigning specific subtype identities based solely on particle size or commonly used isolation methods (31, 32). Exosomes, a subtype of EVs derived from endosomes through the multivesicular body pathway, require appropriate experimental support for definitive classification as “exosomal”, rather than relying solely on size-based criteria (31–33). EVs carry a diverse array of bioactive cargo, including proteins, lipids, and nucleic acids, and their molecular composition is influenced by context-dependent mechanisms of vesicle biogenesis and cargo selection in the originating cells (30, 34).

Notably, ncRNAs can be selectively packaged into EVs and delivered to recipient cells, where they modulate gene expression and cellular function (34). In diabetic retinopathy, the EV-mediated transfer of ncRNAs has been implicated in signaling between retinal endothelial cells, pericytes, glial cells, and inflammatory cells, suggesting a role in coordinating retinal responses to metabolic and vascular stress (10). Consequently, EV-associated ncRNAs may more directly reflect retinal pathological processes than freely circulating molecules, supporting their potential relevance as biologically significant indicators of retinal disease activity (11).

### 3.3 EV-associated ncRNAs as circulating biomarkers

The association of ncRNAs with extracellular vesicles confers several properties that enhance their potential as circulating biomarkers. Encapsulation within EVs protects ncRNAs from enzymatic degradation, resulting in increased stability in biofluids such as serum and plasma (10, 35). Additionally, EV-associated ncRNAs may more accurately reflect disease-relevant cellular processes, as the composition of EV cargo is influenced by changes in the physiological and pathological states of the tissue of origin (30). Compared to freely circulating RNAs, EV-associated ncRNAs may offer improved molecular stability and selective cargo enrichment; however, these advantages can be compromised by variability in EV isolation, purity, and characterization methods (11, 12).

Moreover, EV-associated ncRNAs may capture upstream regulatory alterations involved in the pathogenesis of diabetic retinopathy, rather than merely reflecting downstream consequences of established retinal damage (10). This characteristic is particularly relevant for early disease detection and for monitoring dynamic molecular changes across different stages of DR. Collectively, these features position EV-associated ncRNAs as promising candidates for minimally invasive biomarkers capable of supporting disease detection, stage-specific assessment, and longitudinal tracking of diabetic retinopathy progression (11).

## 4 Network-based regulation of diabetic retinopathy

### 4.1 ceRNA interactions involving miRNAs, lncRNAs, and circRNAs

This section does not aim to catalog ncRNA–target interactions; rather, it seeks to establish a network-based interpretive framework for understanding why stage-specific ncRNA panels, rather than single markers, are necessary for clinically meaningful DR stratification. The ceRNA hypothesis proposes that RNA transcripts containing shared microRNA response elements (MREs) can regulate one another by competing for common miRNAs (36). Within this framework, lncRNAs and circRNAs may function as molecular sponges that sequester miRNAs, thereby modulating the availability of miRNAs to bind their target messenger RNAs (mRNAs) (36, 37). Consequently, changes in the expression of one ncRNA species can propagate through the network and influence entire gene expression programs rather than just single targets (36).

Importantly, the ceRNA framework explains why the same ncRNA may show inconsistent directionality across studies, highlighting the limitations of single-marker approaches and supporting network-based interpretations for stage-specific biomarker development in diabetic retinopathy (38, 39). Within ceRNA networks, the diagnostic significance of a given ncRNA is shaped by its regulatory context rather than its absolute abundance, providing a conceptual basis for interpreting EV-associated ncRNA signatures as network-level biomarkers rather than isolated signals (11). In this sense, apparent variability in circulating miRNA levels across cohorts may reflect differences in upstream lncRNA/circRNA modulation, disease stage, cellular composition, or metabolic environment, rather than true biological contradictions.

In DR, ceRNA interactions are implicated in key pathogenic pathways, including endothelial dysfunction, inflammation, oxidative stress, and pathological angiogenesis (38, 39). miRNAs often serve as the central effector molecules in these networks, directly repressing translation or destabilizing mRNAs involved in vascular permeability and neovascular signaling (9). In contrast, lncRNAs and circRNAs tend to operate upstream, shaping the regulatory landscape by fine-tuning miRNA activity and transcriptional accessibility (24, 28). circRNAs, due to their covalently closed structure, exhibit enhanced stability and may provide sustained miRNA buffering under chronic hyperglycemic stress (24). Notably, ceRNA networks are inherently context- and stage-dependent, underscoring the importance of systems-level interpretation over reductionist single-marker analysis (38).

However, predicted ceRNA relationships should not be equated with experimentally established molecular competition. Functional ceRNA activity relies on relative transcript abundance, shared subcellular localization, and sufficient occupancy of miRNA response elements. Since many DR-associated ceRNA networks are primarily derived from expression correlations or in silico predictions, their application in biomarker panel design should be guided by direct binding, perturbation, and rescue evidence whenever available.

### 4.2 EV-associated ncRNA trafficking and intercellular communication

Beyond intracellular regulation, ncRNAs are involved in intercellular communication by being actively packaged into extracellular vesicles (30). These vesicles protect ncRNAs from enzymatic degradation and facilitate their transport across biological barriers, allowing for long-range signaling between retinal cells and peripheral tissues (10). Consequently, EV-associated ncRNAs may provide a mechanistic link between localized retinal pathology and detectable alterations in circulating biofluids (11).

In DR, EV-mediated ncRNA trafficking may reflect both disease-driven signaling and adaptive responses to metabolic stress. Retinal endothelial cells, pericytes, Müller cells, and infiltrating inflammatory cells can release EVs containing selectively enriched ncRNA cargo, which then modulates angiogenic, inflammatory, and oxidative pathways in recipient cells (8, 40). The selective enrichment of specific ncRNAs within EVs indicates an active sorting mechanism rather than passive cellular leakage, thereby enhancing their biological relevance as disease-related carriers (30, 40).

From a biomarker perspective, EV-associated ncRNAs may provide enhanced molecular stability and may improve the signal-to-noise ratio by enriching disease-relevant cargo compared to freely circulating RNAs; however, this should not be interpreted as evidence of absolute retina specificity. Nonetheless, variability in EV isolation protocols, purification standards, and characterization methods remains a significant translational barrier (11, 12). These technical challenges highlight the need to interpret EV-associated ncRNAs as network-level signals, evaluated under standardized workflows to facilitate decision-support translation, rather than treating them as isolated markers detached from their regulatory and clinical contexts.

### 4.3 Functional relevance to inflammation, oxidative stress, and angiogenesis

The functional convergence of ncRNA networks in DR centers on three interrelated pathogenic axes: inflammation, oxidative stress, and angiogenesis (8, 21). Chronic hyperglycemia induces sustained oxidative stress and low-grade inflammation, which disrupt endothelial homeostasis and promote microvascular damage (2, 18). ncRNAs are embedded within these stress-response circuits, acting as both sensors and modulators of disease progression (8).

miRNAs frequently serve as direct post-transcriptional regulators of inflammatory mediators, antioxidant pathways, and angiogenic factors, enabling rapid adaptive responses (10). In contrast, lncRNAs often influence chromatin architecture, transcription factor recruitment, and epigenetic modification, thereby exerting broader and more sustained regulatory control (28). Emerging evidence suggests that certain lncRNAs intersect with aging-related pathways, including cellular senescence and mitochondrial dysfunction (41, 42). Given that DR is an age-dependent chronic complication, dysregulation of aging-associated lncRNAs may interact with diabetes-driven stress signaling, complicating stage interpretation while reinforcing their upstream regulatory significance (41).

Angiogenesis represents a downstream convergence point where cumulative network dysregulation becomes clinically evident, particularly in advanced stages such as proliferative DR (2). Thus, ncRNA-mediated modulation of angiogenic signaling reflects not just a single molecular alteration but the integrated outcome of prolonged inflammatory and oxidative network perturbations. This systems perspective clarifies why disease severity and progression risk—integrated outputs of prolonged network perturbation—are more accurately captured by coordinated molecular signatures than by any single effector (11).

### 4.4 Rationale for differential focus on ncRNA classes across sections

Although multiple classes of ncRNAs contribute to DR pathogenesis, their translational readiness varies significantly. The preceding subsections primarily discussed lncRNAs and circRNAs within a network-regulatory framework, as their effects are generally indirect, context-dependent, and heavily influenced by epigenetic and aging-related factors (28, 41). These characteristics complicate their interpretation as standalone circulating biomarkers (11).

In contrast, Section 5 focuses on miRNAs as candidates for stage-resolved biomarkers. miRNAs directly regulate mRNA stability and translation, show more consistent detectability in circulating and EV-associated compartments, and often act as final common effectors within ncRNA regulatory networks (10, 12). Their central position enables them to integrate upstream signals from lncRNAs, circRNAs, metabolic stress, and inflammatory pathways. Accordingly, the following section shifts the focus from regulatory architecture to translational evidence, examining how miRNAs—being the most analytically accessible effectors within these networks—function as stage-resolved circulating biomarkers across the DR severity continuum. The network principles established earlier provide the context for understanding why individual miRNA signals may vary across studies and why panel-based, network-informed approaches are ultimately necessary for clinically meaningful stage discrimination (11, 12).

## 5 Stage-resolved miRNA biomarkers in diabetic retinopathy

Stage-resolved analysis of miRNA biomarkers aims to address a critical gap in diabetic retinopathy (DR) by determining how molecular signals are associated with disease severity and whether selected candidates can ultimately support stage discrimination or progression prediction (12). While lncRNAs and circRNAs shape the upstream regulatory context within ceRNA networks, miRNAs serve as central post-transcriptional mediators that integrate these signals, providing the most extensively studied readouts for stage-aware biomarker analysis in diabetic retinopathy (11, 12). In the ncRNA regulatory network, miRNAs play a pivotal role by directly controlling mRNA stability and translation, making them the most analytically mature and clinically actionable class of ncRNAs for stage-resolved biomarker development in diabetic retinopathy (9, 43).

Although many studies continue to categorize DR into NPDR versus PDR, clinical decision-making often relies on earlier inflection points—particularly the transition from mild/moderate NPDR to severe NPDR—where the risk of progression accelerates and follow-up intervals and referral decisions may change (5, 44). However, it is crucial to acknowledge that the existing literature predominantly focuses on broad-category comparisons, such as DR versus no DR or pooled NPDR versus PDR, rather than on detailed adjacent-stage contrasts (12). Primary studies explicitly comparing mild versus moderate NPDR, moderate versus severe NPDR, or severe NPDR versus early PDR remain scarce, and few have been designed with pre-specified adjacent-stage discrimination as the primary endpoint (6, 12). As a result, much of the stage-resolved interpretation presented in this section is inferred from severity-gradient trends, cross-study synthesis, and biological plausibility, rather than derived from direct adjacent-stage evidence.

Readers should interpret the framework below as a translational roadmap that organizes existing signals by clinical use case, while acknowledging that rigorous validation in adjacent-stage cohorts remains an essential and largely unmet requirement (11). For clarity, we distinguish three levels of biomarker evidence throughout this review. “Stage-associated” refers to cross-sectional differences in ncRNA expression or severity-related trends across DR stages without direct evidence of discrimination between predefined stages. “Stage-discriminatory” refers to biomarkers directly evaluated for distinguishing defined clinical stage groups, ideally including adjacent-stage comparisons, with classification or diagnostic-performance measures where available. “Predictive” refers to biomarkers evaluated longitudinally, in which baseline ncRNA levels are associated with subsequent disease progression or another future clinical outcome. Accordingly, cross-sectional severity gradients are considered stage- or severity-associated rather than predictive evidence.

In this section, we synthesize findings on circulating and EV-associated miRNAs within a stage-resolved framework, emphasizing (i) potential use cases (screening versus risk stratification versus progression prediction), (ii) cross-study consistency, and (iii) severity-dependent patterns that may support longitudinal monitoring (11, 12).

### 5.1 miRNAs associated with early DR and mild NPDR (screening biomarkers)

Early DR and mild NPDR represent a biologically active yet clinically subtle phase of the disease. Fundoscopic findings may be limited to minimal microaneurysms or may even appear normal, yet molecular dysregulation is already underway (2, 21). This discrepancy between structural visibility and biological activity presents a critical opportunity for the development of screening-oriented biomarkers (6). To align our interpretation with **Table 4**, we classify early/mild-stage miRNAs into two translational categories: (i) early-stage anchored candidates—miRNAs evaluated in explicit no-DR versus early DR/mild NPDR contrasts or associated with early retinal structural changes—and (ii) supportive candidates—miRNAs that are biologically plausible and frequently dysregulated across DR but are not uniquely specific to early disease. In the text below, anchored candidates are marked in bold with “(anchored)”, while broadly dysregulated candidates are explicitly labeled “(supportive)” to avoid overstating early screening specificity.

**Table 4:** Early-stage miRNA candidates for screening-oriented biomarker development in diabetic retinopathy.

miRNA	Dominant DR stage evaluated	Direction of change	Biofluid/fraction	Validation status	Ref.
Group A. Early-stage–anchored candidates (Direct comparison of no DR vs early/mild NPDR or correlation with early retinal structural changes)					
miR-3976	No DR vs Early NPDR	↑ in early NPDR	Serum-derived EV fraction	Discovery+ validation	(46)
miR-23a-3p	Early DR/mild NPDR vs controls	↓ in early DR	Serum (total circulating RNA)	Single-cohort clinical study; functional support reported	(47)
miR-26a-5p	Early DR (RNFL thinning correlation)	↓ with early structural change	Plasma (total circulating RNA)	Clinical correlation study	(48)
miR-126-5p	Early DR/mild NPDR vs no DR (NDR)	↓ in early DR/mild NPDR	Plasma (total circulating RNA)	Discovery (RNA-seq) + Validation (qRT-PCR)	(48)
Group B. Supportive DR-associated candidates (Reported DR association but without explicit early/mild stage stratification)					
miR-15a-5p	DR vs no DR (NPDR ± PDR mixed)	↓ in DR	Serum (total circulating RNA)	Primary clinical study	(47)
miR-16-5p	DR vs no DR (NPDR ± PDR mixed)	↑ in DR	Plasma-derived EV-enriched fraction	Discovery-level evidence	(53)
miR-93	Control → T2DM → DR (not stage-stratified)	↓ in DR	Serum (total circulating RNA)	Diagnostic performance study	(49)
miR-152	Control → T2DM → DR (not stage-stratified)	↑ in DR	Serum (total circulating RNA)	Diagnostic performance study	(49)
miR-1281	T2DM without DR → DR (NPDR/PDR continuum)	↑ in DR	Serum (total circulating RNA)	Discovery + Validation	(50)
miR-21* *(optional)*	No DR → NPDR → PDR (severity gradient; not early-specific)	↑ with increasing DR severity	Plasma/serum (total circulating RNA)	Two independent primary cohorts	(51, 52)

This table summarizes miRNAs reported in no DR versus early DR/mild NPDR comparisons or related early retinal structural changes, with annotation of directionality, biofluid source, validation status, and translational classification as early-stage anchored or supportive candidates. EV terminology was used preferentially unless exosomal identity was supported by evidence of endosomal biogenesis; terminology used in individual source studies does not necessarily imply definitive EV subtype assignment. *miR-21 is included as a representative severity-associated supportive candidate because it shows progressive association with DR severity, but it should not be interpreted as an early-stage anchored marker for no DR versus early DR/mild NPDR discrimination; ↑, increased; ↓, decreased.

At the biological level, early DR is characterized by endothelial stress, oxidative imbalance, low-grade inflammation, and increased pericyte vulnerability (18, 45). Hyperglycemia-induced reactive oxygen species and NF-κB–mediated inflammatory signaling alter the expression of miRNAs involved in vascular homeostasis and barrier integrity (18, 21). The anchored screening candidates reported in **Table 4** include: miR-3976 (anchored) (46), miR-23a-3p (anchored) (47), miR-26a-5p (anchored) (48), and miR-126-5p (anchored) (48). In contrast, widely reported miRNAs such as miR-93 (supportive) (49), miR-152 (supportive) (49), miR-1281 (supportive) (50), miR-21 (supportive) (51, 52), and the miR-15a/16 family (supportive) (47, 53) are mechanistically linked to endothelial function, VEGF regulation, and apoptotic balance (9). However, these should be interpreted as supportive components unless validated with specific early-stage contrasts.

The transition from early DR to clinically recognizable mild NPDR may represent a functional inflection point rather than a purely structural shift. In early DR, miRNA alterations often reflect inflammatory priming and compensatory endothelial responses aimed at maintaining vascular integrity (21). As the disease progresses toward mild NPDR, there appears to be a shift toward sustained VEGF pathway activation, measurable disruption of tight junction regulation, loss of pericyte–endothelial coupling, and increased vascular permeability (2, 18). This transition reflects a movement from adaptive stress signaling toward structural microvascular destabilization.

For example, miR-126-5p (anchored, but also broadly reported across DR stages), a key regulator of endothelial stability and angiogenic balance (54, 55), was significantly downregulated in the circulating plasma of patients with mild NPDR relative to no-DR controls (48), potentially impairing vascular repair signaling and predisposing them to microaneurysm formation. miR-21 (supportive), associated with inflammatory and fibrotic signaling, demonstrates upregulation in multiple DR contexts and may increase in mild NPDR in some studies (51, 52), consistent with the amplification of microvascular stress rather than strict early-stage specificity. Members of the miR-15a/16 family (supportive), known regulators of VEGF expression, may exhibit severity-dependent shifts across DR (47, 53), suggesting a transition from compensatory angiogenic modulation to maladaptive activation. These examples highlight why biologically compelling candidates should not be claimed as screening biomarkers unless they are “anchored” by explicit early-stage discrimination and reproducible directionality.

Evidence grading and alignment with tables. Findings summarized in **Table 4** reflect early- and mild-stage candidates, with emphasis on the reported stage contrasts, directionality, and validation status. **Table 5** summarizes biological functions, pathways, and biofluid compartments, whereas **Table 1** provides a stage-resolved evidence summary across heterogeneous evidence sources using the review-specific grading framework described in Section 5.5. Anchored candidates are defined by evidence from explicit early-stage comparisons, such as no DR versus early DR/mild NPDR, or by associations with early retinal structural changes. Supportive candidates are prioritized based on recurrent reporting and biological coherence but lack sufficient early-stage specificity. Independent replication and, where feasible, orthogonal validation remain necessary before either category can be translated into screening applications.

**Table 5:** EV-associated and circulating miRNAs in diabetic retinopathy: biological functions, signaling pathways, and biofluid compartments.

miRNA	Clinical context	Direction of change	Major biological function	Key signaling pathway	Sample source/fraction	Evidence type	Ref.
Group A-1. Blood-derived EV-associated miRNAs (serum/plasma EV)							
miR-377-3p	DME vs T2DM	↓ in DME	Anti-angiogenic regulation (VEGF suppression)	VEGF pathway	Serum-derived EV-enriched fraction	Primary clinical/functional study	(62)
miR-3976	No DR vs early DR	↑ in early DR	Retinal ganglion cell survival modulation	NF-κB	Serum-derived EV fraction; serum total RNA also validated	Primary clinical/functional study	(46)
miR-431-5p	PDR vs non-PDR/DM/control	↑ in PDR	Cell adhesion, glucose metabolism modulation	MAPK, RAP1, RAS	Plasma-derived sEV fraction	Primary clinical/functional study	(61)
miR-25-3p	DR vs T2DM	↑ in DR	Endothelial proliferation	NOTCH1, β-catenin	Plasma-derived EV-enriched fraction	Primary clinical study	(53)
miR-320b	DR vs T2DM	↑ in DR	Angiogenic signaling modulation	NOTCH1, β-catenin	Plasma-derived EV-enriched fraction	Primary clinical study	(53)
miR-495-3p	DR vs T2DM	↓ in DR	Loss of anti-angiogenic regulation	NOTCH1 pathway	Plasma-derived EV-enriched fraction	Primary clinical study	(53)
Group A-2. Non-blood EV-associated miRNAs (vitreous/tear EV)							
miR-9-3p	PDR vs control (vitreous)	↑ in PDR	Angiogenesis activation	VEGFR2–AKT/ERK	Vitreous-derived EV-enriched fraction	Primary experimental study	(63)
miR-21-5p	PDR vs control (vitreous)	↑ in PDR	Fibrovascular proliferation	TGF-β, VEGF	Vitreous-derived EV-enriched fraction	Primary clinical study	(64)
miR-125 family	PDR vs control (vitreous)	↓ in PDR	TGF-β pathway dysregulation	TGF-β signaling	Vitreous-derived EV-enriched fraction	Primary clinical study	(64)
miR-145-5p	DR vs T2DM (tear)	↑ in DR	Metabolic stress response	ErbB signaling	Tear-derived sEV fraction	Primary clinical study	(65)
miR-146a-5p	DR vs T2DM (tear)	↓ in DR	Loss of inflammatory regulation	NF-κB	Tear-derived sEV fraction	Primary clinical study	(65)
Group B. Additional DR-related or diabetes-associated circulating miRNAs							
miR-7	T2DM with vs without microvascular complications (MC)	↑ in T2DMC	β-cell dysfunction, insulin dysregulation	mTOR	Serum (total circulating RNA; predominantly EV-free fraction)	Primary clinical study	(66)
miR-126-5p	NDR/mild NPDR vs control	↓ in DR	Endothelial repair impairment, retinal neurodegeneration	PI3K–AKT	Plasma (total circulating RNA)	Discovery + validation (RNA-seq/qRT-PCR)	(48)
miR-200b	PDR-related	↓ in DR	Loss of anti-angiogenic regulation	VEGF	Plasma (total circulating RNA)	Primary clinical study	(67)
miR-29b	DR fibrosis	↓ in DR	ECM remodeling	TGF-β	Plasma (total circulating RNA)	Primary clinical study	(67)
miR-15a-5p	DR vs no DR	↓ in DR	Anti-angiogenic loss → endothelial proliferation	VEGF	Serum (total circulating RNA)	Primary clinical study	(47)
miR-16-5p	DR vs no DR	↑ in DR	Endothelial cell cycle dysregulation	VEGF	Plasma-derived EV-enriched fraction	Primary clinical study	(53)

Group A includes EV-associated miRNAs and is subdivided into two categories: blood-derived EV miRNAs (A-1: serum/plasma EVs) and non-blood-derived EV miRNAs (A-2: vitreous/tear EVs). This distinction highlights differences in clinical accessibility and translational applicability. Blood-derived EV miRNAs are more directly relevant for developing minimally invasive biomarkers, while vitreous- and tear-derived EV miRNAs should primarily be interpreted as mechanistically informative or supportive evidence. Group B encompasses additional DR-associated miRNAs identified predominantly in non-EV circulating biofluids or in fractions that were not characterized as purified EV preparations. Findings from serum, plasma, blood-derived EVs, tears, and vitreous samples should therefore be interpreted within their respective sample compartments and should not be directly compared across matrices. DME is considered a vision-threatening phenotype rather than a sequential stage within the NPDR–PDR continuum; ↑, increased; ↓, decreased.

Clinically, this biological inflection suggests that early DR may represent a potentially reversible or compensatory phase, while mild NPDR marks the point at which microvascular destabilization becomes structurally apparent (2). A screening-oriented miRNA panel should prioritize anchored candidates for discriminating between no DR and early DR/mild NPDR, while using supportive miRNAs to enhance network-level robustness and biological interpretability (**Figure 1**) (11, 12). Such a panel could identify patients transitioning from molecular stress to structural damage, stratify follow-up intensity before progression to moderate NPDR, and complement imaging-based screening by capturing biologically active disease that is not yet fully expressed in structural findings.

**Figure 1:**
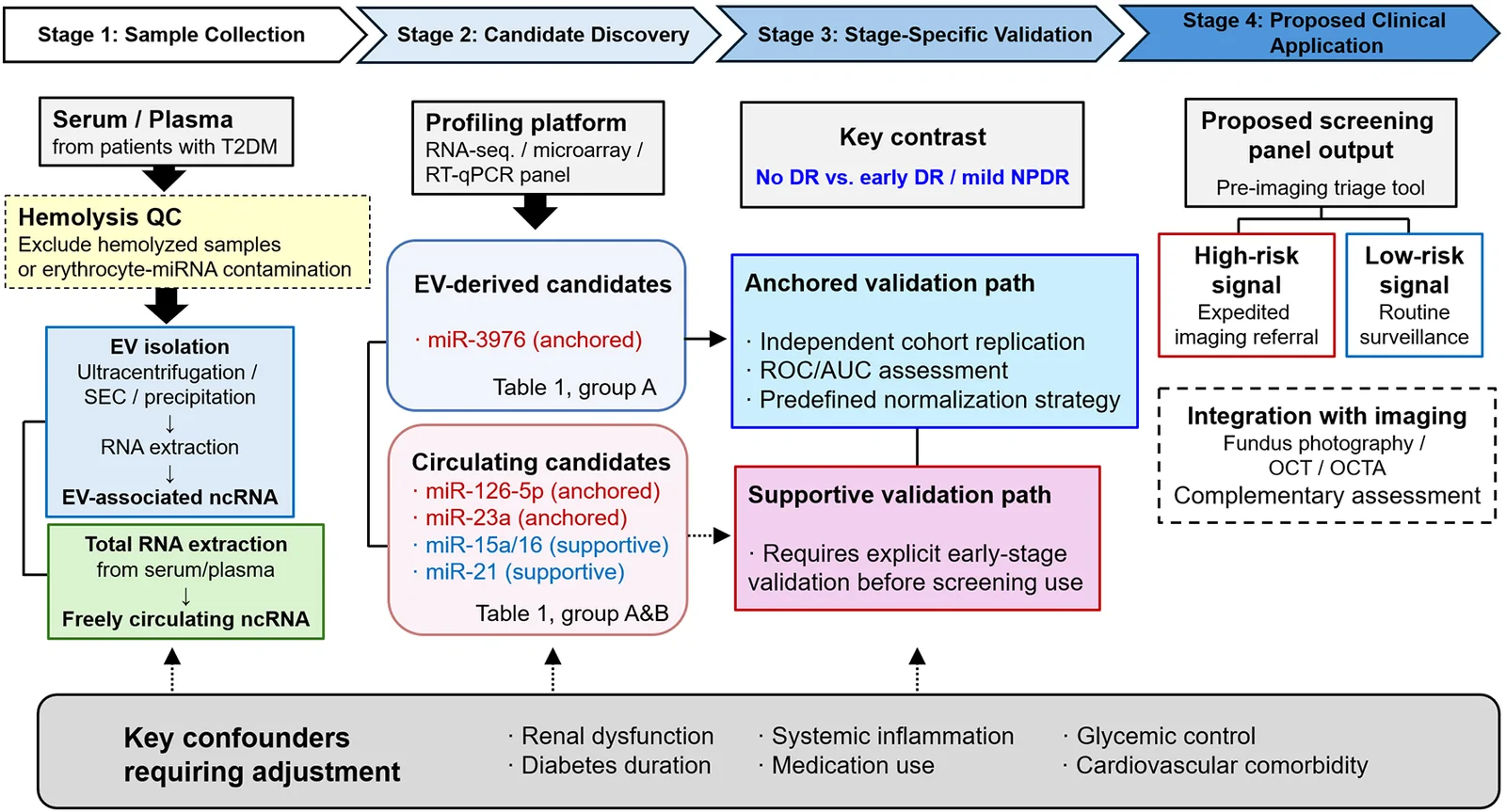
Conceptual pipeline for screening-oriented ncRNA biomarker development in early diabetic retinopathy. This schematic outlines a four-stage workflow for developing circulating ncRNA biomarkers that target the transition from no diabetic retinopathy (no DR) to early diabetic retinopathy/mild non-proliferative diabetic retinopathy (NPDR). Serum or plasma samples first undergo pre-analytical quality control, which includes hemolysis assessment, followed by the analysis of extracellular vesicle (EV)-associated and freely circulating ncRNA fractions. Candidate miRNAs are then classified as early-stage anchored candidates, supported by explicit early-stage contrasts, or as supportive candidates with broader DR-related dysregulation but limited early-stage specificity. Representative anchored candidates include miR-3976, miR-23a-3p, and miR-126-5p, while supportive candidates include miR-21 and the miR-15a/16 family. Anchored candidates require independent validation with ROC/AUC assessment for discrimination between no DR and early DR/mild NPDR. A validated panel may serve as a pre-imaging triage tool to prioritize high-risk patients for expedited ophthalmic imaging, complementing standard fundus photography, OCT, or OCTA. The clinical application shown in Stage 4 represents a proposed translational use and has not yet been prospectively validated as a clinical decision pathway.

### 5.2 miRNAs associated with moderate NPDR and risk stratification

Moderate NPDR represents a biologically unstable and clinically heterogeneous phase of diabetic retinopathy. While some patients remain stable, others progress toward severe NPDR or proliferative disease within a relatively short interval (12). At this stage, the clinical focus shifts from simple detection to risk stratification—identifying patients whose retinal microenvironment is transitioning toward ischemia-driven progression. Pathophysiologically, moderate NPDR reflects expanding capillary nonperfusion, progressive endothelial dysfunction, and emerging hypoxic stress (44). Unlike early DR, which is dominated by inflammatory priming and compensatory vascular responses, moderate NPDR begins to exhibit sustained activation of hypoxia-related signaling pathways, including VEGF upregulation and increased vascular permeability (2). Thus, the most informative miRNAs at this stage are those associated with ischemic burden, endothelial destabilization, and inflammatory amplification, rather than markers that merely reflect long-standing systemic diabetes severity.

In our stage-resolved evidence table (**Table 1**), several candidate miRNAs identified in moderate NPDR cohorts align with these mechanisms. For example, miR-21 has been linked to persistent inflammatory signaling and microvascular remodeling (52). At the moderate NPDR stage, serum miR-21 shows a significant increase compared to the mild stage, consistent with its role in NF-κB-mediated endothelial dysfunction and VEGF-responsive amplification (9, 52). Meanwhile, miR-34a reflects oxidative stress-related endothelial vulnerability; its expression decreases progressively from mild to moderate and severe NPDR, mirroring the gradual exhaustion of the SIRT1-dependent antioxidant axis (52, 56). This inverse pattern—rising miR-21 and falling miR-34a at the moderate stage—may serve as a complementary expression signature reflecting the transition from compensated to decompensated vascular homeostasis. Members of the miR-15/16 family, known regulators of VEGF expression and retinal endothelial barrier integrity (57), may demonstrate severity-dependent shifts consistent with the transition toward hypoxia-driven angiogenic priming. Indeed, reduced serum miR-15a-5p has been reported in both diabetic patients without DR and those with early-to-moderate NPDR, with a further decline as retinopathy advances (47). When interpreted within a severity-aligned framework, these miRNAs cluster around pathways of ischemia and vascular instability.

Because moderate NPDR sits at the intersection between screening-stage disease and advanced angiogenic transformation, single-marker approaches may be insufficient (12). Multi-marker panels integrating inflammation, oxidative stress, and endothelial dysfunction pathways may better capture the multidimensional dysregulation characteristic of this stage. Such an approach supports the rationale for network-based panel strategies discussed in Section 8, where biological coherence and clinical robustness are central considerations.

### 5.3 miRNAs associated with severe NPDR and progression-related biomarkers

Severe NPDR represents a critical biological and clinical threshold in diabetic retinopathy, as it is strongly associated with the near-term conversion to proliferative DR (PDR) and vision-threatening complications. At this stage, a major objective of biomarker research is to identify molecular signals that may ultimately support prediction of progression to overt neovascularization. However, such predictive utility requires longitudinal validation rather than cross-sectional stage association alone. Natural history data from the ETDRS and subsequent real-world cohorts indicate that eyes with severe NPDR carry a 1-year risk of PDR conversion of approximately 50%, which rises to over 75% for very severe NPDR. This underscores the clinical urgency of developing predictive biomarkers at this stage (58).

Pathophysiologically, severe NPDR is characterized by extensive capillary dropout, marked nonperfusion, escalating hypoxic stress, and robust activation of the HIF–VEGF axis (2). Unlike earlier stages dominated by inflammatory priming or emerging ischemia, severe NPDR reflects a retina approaching the angiogenic tipping point. Persistent endothelial destabilization, increased vascular permeability, and amplification of pro-angiogenic signaling create a feed-forward loop that predisposes the retina to neovascular conversion (44). Accordingly, the most compelling miRNA candidates at this stage are those demonstrating severity-dependent behavior—monotonic changes across mild → moderate → severe NPDR—and/or predictive signals in longitudinal cohorts.

In our stage-resolved evidence table (**Table 1**), several miRNAs exhibit patterns consistent with this biology. For instance, the progressive upregulation of miR-21 across advancing NPDR stages aligns with sustained inflammatory amplification and vascular remodeling. Serum miR-21 levels show a graded increase from normal fundus through mild, moderate, and severe NPDR to PDR, positioning it as a severity-tracking marker rather than merely a stage-discriminating one (9, 52). miR-34a, associated with oxidative stress and endothelial apoptosis, may reflect cumulative microvascular injury in severe NPDR. Its progressive decline across advancing stages—mediated through SIRT1 suppression and mitochondrial dysfunction in retinal vascular endothelial cells—tracks the exhaustion of cytoprotective reserves as the proliferative threshold approaches (52, 56). Severity-dependent shifts in VEGF-regulatory miRNAs, such as the miR-15/16 family—which target VEGF and TGF-β3 to maintain endothelial barrier integrity—suggest a transition from compensatory angiogenic modulation to maladaptive hypoxia-driven signaling (47, 57). When interpreted along the severity axis, these miRNAs cluster around ischemia, endothelial failure, and angiogenic priming—mechanisms central to the imminent development of PDR (12).

A key challenge, however, is that many circulating miRNAs also reflect systemic inflammation, generalized vascular injury, or comorbid cardiovascular disease, potentially diluting retinal specificity (6). EV-associated fractions may partially mitigate this limitation by enriching for disease-relevant cargo and improving molecular stability (10). Nonetheless, claims of retinal relevance require triangulation with ocular samples, mechanistic validation in retinal cell models, or correlation with imaging-derived ischemic metrics (12). For predicting severe NPDR progression, future studies should prioritize granular staging (explicitly categorizing mild, moderate, and severe NPDR rather than using pooled categories), pre-specified clinical endpoints such as time-to-PDR or treatment escalation, and standardized analytical pipelines that include hemolysis assessment and normalization controls (12). Only through severity-aligned and longitudinally designed investigations can miRNA signatures evolve from associative markers into clinically actionable predictors of proliferative conversion (44).

### 5.4 miRNAs associated with PDR and advanced disease validation

Proliferative diabetic retinopathy (PDR) represents the culmination of progressive retinal ischemia, characterized by pathological neovascularization driven by sustained hypoxia and heightened inflammatory signaling. At this stage, the HIF–VEGF axis is significantly activated, endothelial instability is pronounced, and angiogenic remodeling prevails in the retinal microenvironment (2). Consequently, PDR-associated miRNAs are often easier to detect and exhibit larger effect sizes compared to early-stage signals, particularly in comparisons between PDR and non-DR or pooled NPDR cohorts (12).

Biologically, PDR reflects a transition from ischemic priming to overt angiogenic execution. miRNAs implicated at this stage frequently cluster around pathways involved in VEGF signaling, extracellular matrix remodeling, endothelial proliferation, and inflammatory amplification (9). In our stage-resolved table (**Table 1**), several miRNAs demonstrate severity-aligned escalation patterns consistent with this biology. For instance, the progressive elevation of miR-21 corresponds with sustained inflammatory activation and fibrotic remodeling in advanced DR—miR-21 is significantly elevated in the vitreous humor of PDR eyes with active fibrovascular membranes and is induced by TGF-β, a key driver of fibrogenesis in PDR (59). This is further supported by a three-miRNA serum panel comprising miR-21, miR-181c, and miR-1179, which achieved an AUC of 0.89 for distinguishing PDR from NPDR across two independent validation sets (60). Meanwhile, oxidative stress–associated miRNAs such as miR-34a may indicate cumulative endothelial injury at the PDR stage (52). Alterations in VEGF-regulatory miRNAs, including members of the miR-15/16 family, may signify the breakdown of earlier compensatory control and the establishment of a pro-angiogenic state (57). Notably, serum EV-associated miR-431-5p has been reported to be elevated approximately two-fold in patients with PDR compared to healthy controls and diabetic patients without PDR, with functional evidence suggesting it promotes retinal capillary endothelial cell proliferation; therefore, it is best interpreted as a PDR-stage, EV-associated biomarker candidate rather than an early-stage screening marker (61).

However, advanced disease validation should not be conflated with the utility of early detection. A miRNA that effectively distinguishes PDR from no DR may primarily reflect late-stage angiogenic amplification rather than clinically actionable transition points. From a translational standpoint, the more challenging—and clinically meaningful—comparison lies between severe NPDR and early PDR, where the biological boundary between ischemic stress and active neovascularization is most relevant to management decisions. This gap is underscored by meta-analytic data showing that the highest diagnostic accuracy for circulating miRNAs in DR was observed in the NPDR vs. PDR comparison (pooled AUC 0.90), with upregulated miRNAs achieving the highest diagnostic odds ratio of 36.32 (12).

Robust validation in PDR requires replication across independent cohorts, stage-specific comparisons, and careful evaluation of confounding factors such as systemic hypoxia, renal dysfunction, and generalized inflammatory states, all of which may influence circulating miRNA levels (6). EV-associated fractions may enhance biological relevance by enriching actively secreted angiogenic cargo; however, claims of retinal specificity necessitate mechanistic corroboration or correlation with retinal imaging metrics (10). When appropriately framed, PDR-stage miRNAs retain potential clinical value. Rather than serving solely as detection markers, they may contribute to progression monitoring, assessment of angiogenic activity or treatment response, and multi-marker panels designed to differentiate late NPDR from early PDR (44). Within a stage-aware framework, advanced disease miRNAs thus represent not the endpoint of biomarker discovery but a biologically validated anchor that strengthens the development of network-based panels.

### 5.5 Cross-study consistency and severity-dependent expression patterns

Given the heterogeneity in profiling platforms and study designs, interpreting stage-resolved miRNA literature necessitates a structured consistency framework. Candidate miRNAs were qualitatively evaluated across four domains: (i) the number of independent supporting studies, (ii) agreement in the direction of expression change, (iii) stage specificity and granularity of the reported comparison, and (iv) the availability of independent validation, severity-dependent trends, or longitudinal/predictive evidence. This evidence-grading framework was designed as a pragmatic, review-specific approach for prioritizing reproducible and stage-relevant signals rather than as a formal GRADE assessment or quantitative meta-analysis.

Evidence was classified as low, moderate, or high. Low evidence was assigned primarily to candidates supported by a single primary study, without independent replication, or with limited stage resolution. Moderate evidence was assigned when a candidate was supported by multiple independent studies and/or independent validation with generally consistent directionality, but direct discrimination between clinically relevant adjacent stages or longitudinal predictive validation remained limited. High evidence was reserved for candidates supported by multiple independent cohorts with consistent directionality, explicit clinically relevant stage discrimination, and robust independent and/or longitudinal validation. Candidates were not upgraded solely on the basis of biological plausibility or statistical significance in a single study.

Severity-dependent patterns are particularly important because they align with real-world clinical needs: identifying the transition from mild/moderate NPDR to severe NPDR and forecasting progression to PDR. In practice, severity dependence may manifest as gradual monotonic changes across stages or as threshold-like shifts around severe NPDR or early PDR (52, 53). However, apparent inconsistencies may arise from differences in biofluids (serum vs. plasma), EV isolation and characterization, normalization strategies, and patient-level confounders (12). Therefore, synthesis should emphasize candidates that remain directionally stable across methodological variations and demonstrate reproducible associations with stage granularity (**Figure 2**).

**Figure 2:**
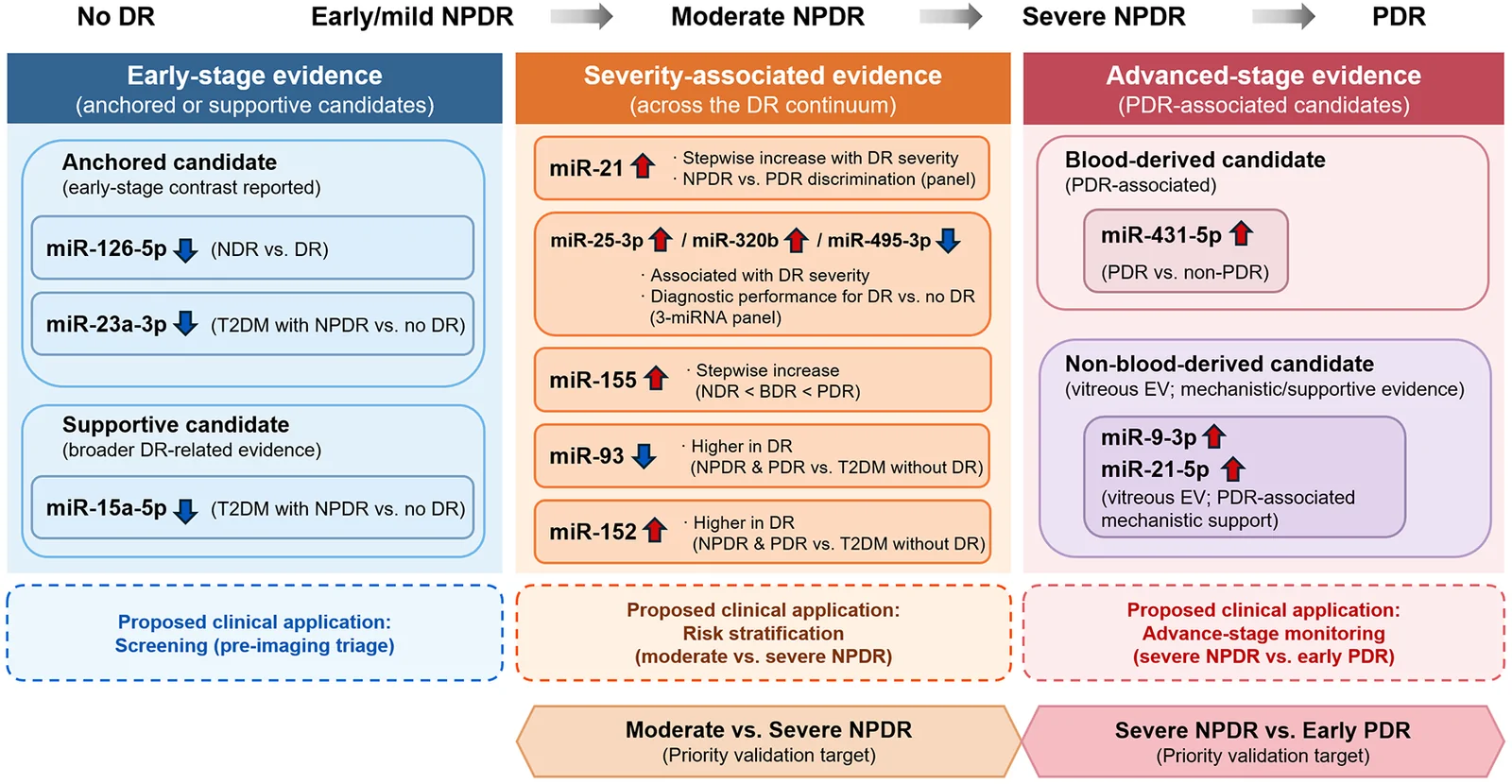
Stage-aware mapping of current miRNA evidence and proposed clinical applications across the diabetic retinopathy severity continuum. Candidate placement reflects the disease stages, stage contrasts, or severity-related patterns evaluated in the underlying studies and does not necessarily indicate validated discrimination between adjacent DR stages. Evidence-supported associations are distinguished from proposed clinical applications, including screening, risk stratification, and progression/advanced-disease monitoring. Early-stage anchored candidates are supported by explicit early-stage contrasts, whereas supportive and severity-associated candidates have broader DR-related or cross-sectional evidence with more limited stage specificity. Advanced-stage candidates include blood-derived EV-associated biomarkers and non-blood EV-associated signals that primarily provide supportive or mechanistic evidence. Proposed clinical applications require independent validation in clinically relevant adjacent-stage comparisons. In particular, moderate versus severe NPDR and severe NPDR versus early PDR are highlighted as priority validation targets. Directionality and biofluid source are indicated where relevant.

An additional source of heterogeneity that warrants explicit acknowledgment is the biofluid compartment from which ncRNAs are measured. This review primarily focuses on blood-based (serum/plasma) circulating and EV-associated ncRNAs as minimally invasive biomarkers. However, several candidates listed in **Table 5**—including miR-9-3p, miR-21-5p, and miR-125 family members from vitreous humor (63, 64), and miR-145-5p and miR-146a-5p from tear fluid (65)—were identified in non-blood biofluids. These sources differ fundamentally in terms of clinical accessibility, collection standardization, and biological proximity to retinal pathology (10). While vitreous-derived signals are mechanistically informative, they require invasive sampling and are therefore unsuitable for routine screening or longitudinal monitoring (11). In contrast, tear-derived ncRNAs allow for non-invasive collection but encounter distinct pre-analytical challenges, including variable collection methods and low RNA yield (65). Accordingly, non-blood-derived candidates in **Table 5** (Group A-2) should be interpreted as mechanistically supportive evidence rather than direct screening or monitoring biomarker candidates, and their findings should not be conflated with the performance of blood-based biomarkers in stage-resolved analyses.

## 6 Stage-associated lncRNAs and circRNAs in DR progression

Unlike miRNAs, current evidence does not support lncRNAs or circRNAs as reliable early-stage biomarkers in DR. Instead of serving as primary screening tools, these RNA classes are better positioned as advanced-stage-associated or context-defining regulators within stage-aware biomarker frameworks. Therefore, this section focuses on defining their roles in progression monitoring and network-based panels, rather than overstating their diagnostic readiness.

### 6.1 lncRNAs implicated in advanced and progressive DR

From a clinical perspective, the most critical phase of diabetic retinopathy (DR) progression is not only the transition from non-proliferative DR (NPDR) to proliferative DR (PDR) but also the progression within NPDR itself, particularly toward severe NPDR, which precedes overt neovascularization. However, compared to miRNAs, the current evidence supporting circulating long non-coding RNAs (lncRNAs) as stage-resolved biomarkers remains limited. Most available studies compare NPDR and PDR or DR versus non-DR groups without explicitly stratifying mild, moderate, and severe NPDR.

Among the reported candidates, PPT2-EGFL8, identified in serum-derived extracellular vesicles (EVs), showed decreased expression in PDR compared to non-PDR and diabetic patients without retinopathy (69). This change was accompanied by increased levels of miR-423-5p, suggesting a ceRNA-related regulatory axis associated with hypoxia-driven angiogenic signaling (69). Although NPDR sub-stage stratification was not performed, the EV-based design and inclusion of multiple clinical groups support the classification of PPT2-EGFL8 as an advanced-stage-associated circulating candidate rather than an early screening marker.

Similarly, NR2F1-AS1 has been evaluated in cohorts that include healthy individuals, diabetic patients without DR, NPDR, and PDR. Increased expression correlated with more advanced disease stages, and the study emphasized stage-related discrimination (70). While currently supported by a single primary investigation, the observed severity-dependent pattern enhances its relevance to advanced-stage transition. Additional serum lncRNAs—including MIAT, HOTTIP, and SNHG16—have shown elevated expression in PDR compared with NPDR (71). However, these findings derive from single cross-sectional cohorts without detailed severity stratification within NPDR. Consequently, these lncRNAs are best interpreted as signals associated with advanced stages, requiring independent replication and longitudinal validation.

Overall, circulating lncRNAs in DR predominantly demonstrate associations with advanced or proliferative stages. Robust evidence supporting discrimination between adjacent stages—particularly moderate versus severe NPDR—remains insufficient, limiting their current use as independent stage-resolved biomarkers.

### 6.2 circRNAs as stable biomarkers of advanced and progressive DR

Circular RNAs (circRNAs) have a covalently closed structure that enhances their resistance to exonuclease-mediated degradation, making them promising candidates for circulating biomarkers. However, compared to miRNAs, the clinical literature evaluating circRNAs in diabetic retinopathy (DR) is still limited in both scale and stage resolution.

Most published studies focus on advanced or proliferative disease. For instance, hsa_circ_0001953 was found to be elevated in whole blood in proliferative diabetic retinopathy (PDR) compared with NPDR and healthy controls, supported by validation and ROC-based performance assessment (72). While this indicates its association with advanced disease, the lack of NPDR sub-stage stratification and longitudinal evaluation limits its applicability for predicting early progression. Emerging evidence from extracellular vesicle (EV)-based investigations has introduced a broader representation of disease stages. circMKLN1, identified in serum-derived EVs, was evaluated across control individuals, diabetic patients without DR, and NPDR and PDR groups, showing progressively increased expression with disease severity, with the highest levels observed in PDR (73). Although this design improves stage contextualization, comparisons between adjacent stages—particularly within NPDR subcategories—are still insufficiently characterized. Additional serum EV-associated profiling studies have reported dysregulated circRNA panels in PDR, including circFndc3b (hsa_circ_0006156) (74). These studies highlight the detectability and potential angiogenic relevance of circRNAs; however, they remain predominantly exploratory, cross-sectional, and limited to comparisons of advanced stages.

A broader structural limitation must be acknowledged: the total number of circulating circRNA studies in Diabetic Retinopathy (DR) is small, cohort sizes are modest, and methodological heterogeneity—particularly in back-splice junction detection and quantification pipelines—complicates cross-study reproducibility. Consequently, current circRNA evidence is best interpreted as early-stage translational exploration rather than as established stage-resolved biomarker validation.

Overall, circRNAs in DR currently show a dominant association with advanced or proliferative stages. Evidence supporting fine-grained discrimination within NPDR or robust longitudinal prediction remains limited, highlighting the need for larger, severity-stratified, and imaging-integrated validation cohorts. At this stage of evidence, circRNAs are most appropriately recommended as supportive, context-defining components within multi-ncRNA panels—rather than as standalone biomarkers for stage-specific decision support.

### 6.3 Comparison of miRNA-, lncRNA-, and circRNA-based stage discrimination

When non-coding RNAs are evaluated within a stage-resolved framework of diabetic retinopathy (DR), significant differences emerge in both evidentiary strength and functional positioning. Currently, miRNAs provide the most consistent evidence for stage-associated expression patterns, with selected candidates also showing stage-discriminatory performance. Reported candidates range from early DR/mild NPDR to severe NPDR and PDR, with several showing severity-aligned expression trends (75). Although comparisons between adjacent stages within NPDR are often underpowered in many studies, miRNAs are the only class with recurring signals across independent cohorts and multiple biofluids (76). Their central role as post-transcriptional effectors, combined with their relative analytical maturity, supports their status as primary candidates for stage discrimination.

In contrast, circulating lncRNAs and circRNAs are more frequently reported in advanced-stage comparisons, typically NPDR versus PDR or DR versus no DR. Stratification across mild, moderate, and severe NPDR remains limited, and most studies are cross-sectional (76). Consequently, current evidence does not support these RNA classes as standalone early-stage biomarkers or reliable discriminators between adjacent stages. However, these differences reflect not only the volume of studies but also their biological roles. miRNAs act as rapid effector regulators of inflammatory, oxidative, and angiogenic pathways, making them more likely to show measurable shifts during early microvascular stress (77). In contrast, lncRNAs and circRNAs primarily function as upstream modulators within ceRNA networks, influencing transcriptional programs and miRNA availability (75). Their alterations may therefore become more apparent in later stages characterized by ischemia, hypoxia signaling, and broader regulatory reorganization.

In summary, miRNAs, lncRNAs, and circRNAs should not be viewed as competing biomarker classes. Instead, they occupy complementary positions within a hierarchical, network-informed framework: miRNAs represent the most mature stage-associated biomarker class, with selected candidates showing stage-discriminatory potential, while lncRNAs and circRNAs provide context-defining regulatory layers (76). This interpretation supports their integration within structured, stage-specific multi-ncRNA panels rather than using them as isolated markers (75).

## 7 Interpretation of stage-specific ncRNA biomarkers

### 7.1 Influence of biofluid type and EV isolation on stage-specific signals

Interpretation of stage-specific non-coding RNA (ncRNA) biomarkers in diabetic retinopathy (DR) is significantly influenced by pre-analytical variables, particularly the sample source and extracellular vesicle (EV) isolation strategies. In this article, “EV” is used as the preferred umbrella term. The term “exosome” is retained only when used in the original study or when endosomal biogenesis is experimentally supported; otherwise, the reported particles are referred to as EVs or small EVs. Importantly, EV-associated ncRNAs are generally more resistant to RNase-mediated degradation than freely circulating RNAs due to their encapsulation within lipid bilayers (10). This structural protection offers a key advantage for biomarker development by enhancing RNA stability and improving analytical reproducibility across samples and platforms.

Circulating ncRNAs have been measured from whole blood, plasma, serum, and isolated EV fractions, each capturing distinct RNA populations. Accordingly, findings obtained from serum and plasma should be interpreted separately rather than treated as directly interchangeable, because differences in sample preparation and matrix composition may influence measured ncRNA abundance. Likewise, total circulating ncRNAs and blood-derived EV-associated ncRNAs represent distinct analytical compartments and should not be directly compared. Tear- and vitreous-derived ncRNAs should also be interpreted separately from blood-based biomarkers because they differ in clinical accessibility, collection characteristics, and biological proximity to retinal pathology. EV-bound ncRNAs are increasingly favored because they may preferentially reflect signals from their cell of origin, including those derived from retinal tissues, while free ncRNAs may be more susceptible to systemic confounders such as inflammation or metabolic status.

However, EV isolation methods—including ultracentrifugation, polymer-based precipitation, and size-exclusion chromatography—vary significantly in yield, purity, and EV subtype enrichment (78). These methodological differences can substantially affect ncRNA abundance and composition, thereby influencing reported stage-specific associations (78). EV characterization is similarly heterogeneous across studies, with variable use of particle sizing/counting, EV-enriched protein markers, and assessment of non-EV contaminants. Importantly, particle size or the detection of commonly used EV markers alone does not establish endosomal biogenesis and therefore does not justify definitive classification as exosomes (32). Consequently, EV-associated ncRNA findings should be interpreted in the context of both the isolation method and the extent of vesicle characterization reported in each study. As few studies directly compare isolation techniques within the same cohort, discrepancies across studies should be interpreted with caution.

Therefore, expression levels and diagnostic performance reported across serum, plasma, blood-derived EVs, tears, and vitreous samples should not be considered directly comparable without matrix-specific validation.

### 7.2 Biological plausibility of blood-based ncRNA biomarkers for retinal disease

A key challenge in interpreting blood-based ncRNA biomarkers for DR lies in establishing the biological plausibility that retinal pathology can be inferred from peripheral circulation. This concern is particularly relevant given the presence of the blood–retinal barrier (BRB), which restricts molecular exchange between the retina and systemic circulation (79). However, emerging evidence suggests that retina-derived extracellular vesicles can cross a compromised BRB and enter the bloodstream under pathological conditions such as inflammation, ischemia, and neovascularization (10). Experimental and clinical studies have reported increased EV trafficking across the BRB in DR, supporting the notion that circulating EV-associated ncRNAs may, at least in part, reflect retinal disease states (79).

Nevertheless, this process is unlikely to be uniform across disease stages. BRB disruption is more pronounced in advanced DR, particularly in severe NPDR and PDR, which may partly explain why many circulating ncRNA studies preferentially identify biomarkers associated with later stages of the disease (10). However, their interpretability may vary depending on disease severity and BRB integrity, underscoring the need for careful stage-specific analysis.

### 7.3 Stage definition, clinical relevance, and limitations of current evidence

Most existing ncRNA biomarker studies classify diabetic retinopathy (DR) into NPDR versus PDR—a pathologically clear distinction but one that is often insufficient for early intervention and decision support in clinical practice. Clinically, a key inflection point exists within NPDR itself, particularly in the transition from mild/moderate NPDR to severe NPDR. This transition precedes proliferative conversion and is associated with a significantly increased risk of vision-threatening complications (80). However, relatively few biomarker studies stratify NPDR by severity with adequate granularity, limiting the identification of ncRNA signals that could predict imminent progression.

This limitation complicates the interpretation of “stage-specific” findings. Biomarkers derived from comparisons between NPDR and PDR may predominantly reflect late-stage biology rather than early progression risk, thus providing limited utility for screening or early risk stratification. Additionally, expression differences between adjacent stages (e.g., moderate vs. severe NPDR) are often modest and can be obscured by technical variability, heterogeneous analytical pipelines, and small cohort sizes (76). As a result, current evidence supports ncRNAs primarily as indicators of disease presence or advanced-stage disease, while robust biomarkers for early transition within NPDR remain an unmet need.

Taken together, these considerations highlight the necessity for severity-stratified cohorts, standardized pre-analytical and analytical workflows, and longitudinal, imaging-integrated study designs. Addressing these gaps is essential to advance ncRNA biomarkers from descriptive associations to clinically actionable tools that identify high-risk patients at the stage when intervention is most effective. The key interpretive considerations for stage-specific ncRNA biomarker development are summarized in **Box 1**.

Box 1 **Key interpretive considerations for stage-specific ncRNA biomarkers in diabetic retinopathy.**

- EV-associated ncRNAs provide biologically plausible circulating readouts of DR-related processes, but their detectability and interpretability may be stage-biased, particularly toward severe NPDR and PDR where blood–retinal barrier disruption is more pronounced (10).

- Pre-analytical variability, including sample type, hemolysis, EV isolation/characterization, and platform-dependent quantification, substantially contributes to cross-study inconsistency in reported ncRNA signatures (78, 81).
- Among ncRNA classes, miRNAs currently offer the strongest translational readiness for stage-resolved biomarker development, whereas lncRNAs and circRNAs are best interpreted as complementary, network-informing components rather than standalone diagnostic markers (75).
- A major unmet need is the identification of biomarkers that capture clinically actionable transition points, especially adjacent-stage shifts within NPDR, such as moderate to severe NPDR, and the severe NPDR–early PDR boundary (80).
- Progress toward clinical decision-support translation will require severity-stratified and longitudinal cohorts, imaging-integrated endpoints, and standardized analytical pipelines enabling reproducible, externally validated panel performance (76).

## 8 From single biomarkers to stage-specific network-based panels

### 8.1 Limitations of single ncRNA biomarkers

Single circulating ncRNAs—most commonly individual miRNAs—often show statistically significant associations with diabetic retinopathy (DR); however, their clinical applicability is limited when transitioning from broad case–control discrimination to stage-resolved decision-making. Across studies, expression patterns frequently exhibit inconsistencies due to variations in cohort composition, sample processing, normalization strategies, and systemic disease burden. Importantly, many of these “inconsistencies” are not true biological contradictions but rather reflect differences in the measured compartment (freely circulating vs. EV-associated), the applied stage boundaries, and the systemic conditions that co-vary with advanced diabetes. More fundamentally, DR is a multifactorial, network-driven microvascular complication in which inflammation, oxidative stress, hypoxia signaling, endothelial dysfunction, and neurovascular alterations interact dynamically across stages. Circulating ncRNA levels may also be influenced by systemic and clinical confounders, including age, renal dysfunction, glycemic control, systemic inflammatory status, diabetes duration, and medication use, several of which co-vary with advanced diabetes and can either mimic or mask retina-relevant signals. Consequently, single ncRNA biomarkers may perform reasonably well in “PDR vs. no DR” comparisons yet still fail in clinically critical adjacent-stage contrasts (e.g., mild vs. moderate NPDR, moderate vs. severe NPDR, or severe NPDR vs. early PDR), where effect sizes are smaller and confounding is stronger. These limitations suggest that, while biologically meaningful, isolated ncRNAs represent only partial reflections of a broader regulatory network; thus, single-marker strategies are inherently constrained in DR, whereas network-informed panels are better suited to capture stage-dependent biology amid real-world variability.

### 8.2 ceRNA- and EV-associated multi-ncRNA panels

A network-based framework offers a more biologically coherent strategy for biomarker development. Within the ceRNA regulatory architecture, miRNAs serve as central post-transcriptional regulators, directly modulating angiogenic, inflammatory, and hypoxia-responsive genes (77). lncRNAs and circRNAs act as competitive endogenous RNAs, buffering or fine-tuning miRNA activity and providing complementary, context-defining regulatory information (76). Integrating these ncRNA classes into multi-component panels captures coordinated regulatory dynamics rather than isolated molecular fluctuations. Additionally, extracellular vesicles (EVs) serve as stabilizing carriers that protect ncRNAs from degradation and reflect intercellular communication between retinal tissue and systemic circulation (10). Therefore, EV-associated multi-ncRNA panels are not merely additive biomarker combinations but structured representations of disease-relevant regulatory networks, where miRNAs are central, lncRNAs and circRNAs are modulatory, and EVs function as biological stabilizers (**Figure 3**) (75).

**Figure 3:**
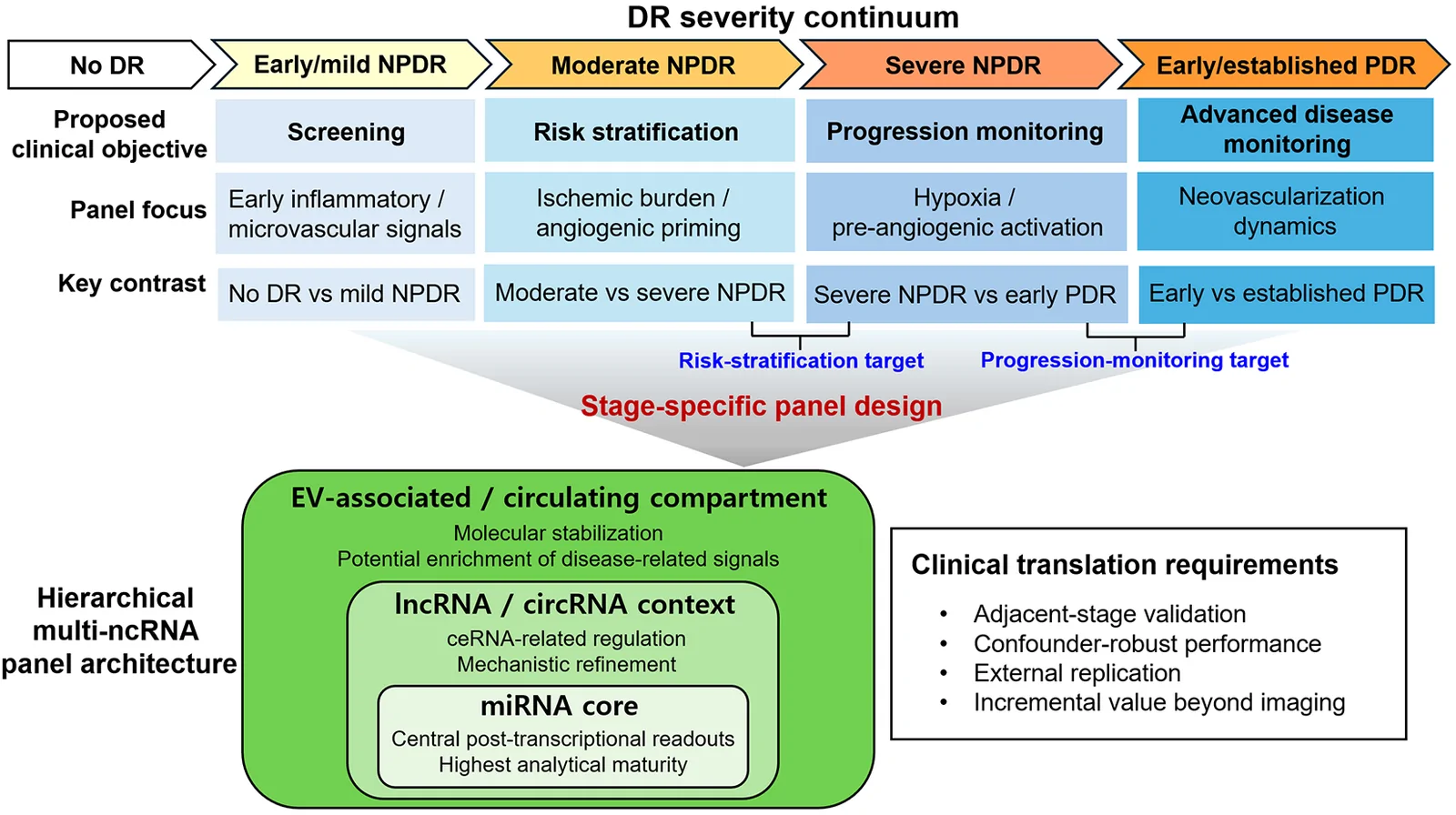
Stage-aware framework for circulating ncRNA biomarker panels in diabetic retinopathy. The upper portion depicts the diabetic retinopathy (DR) severity continuum, ranging from no DR to proliferative DR (PDR), and links each stage to a primary clinical objective: screening, risk stratification, or progression monitoring. These clinical objectives represent proposed translational applications rather than validated clinical uses. Corresponding panel focuses include early inflammatory and microvascular signals, ischemic burden and angiogenic priming, hypoxia-associated pre-angiogenic activation, and neovascularization dynamics. Clinically relevant contrasts between adjacent stages, such as no DR versus mild NPDR, moderate versus severe NPDR, severe NPDR versus early PDR, and early versus established PDR, are highlighted as key validation targets. The lower portion illustrates a hierarchical, network-informed architecture for multi-ncRNA panels. miRNAs serve as central post-transcriptional readouts, exhibiting the strongest analytical maturity and clinical replication. lncRNAs and circRNAs provide contextual regulatory information through ceRNA-related interactions, regulatory buffering, and mechanistic refinement. The EV-associated compartment contributes to molecular stabilization, protection from degradation, and potential enrichment of disease-related circulating signals. Clinically actionable panels should integrate miRNA-centered signals with network-level regulatory context, demonstrating robust performance against confounders and providing incremental value beyond imaging-based assessments.

### 8.3 Integrated strategies for screening and progression tracking

Panel development should be stage-specific rather than quantitatively expansive. Simply increasing the number of ncRNAs does not guarantee better clinical performance; instead, panels should be purpose-built for distinct clinical questions and aligned with stage-specific decision points. Early DR/mild NPDR screening panels should prioritize sensitivity to subtle microvascular and inflammatory changes that precede overt structural damage (77). In moderate NPDR, risk-stratification panels should focus on signals associated with ischemic burden and early angiogenic priming, aiming to identify patients likely to transition to severe NPDR (80). For severe NPDR and PDR, progression-monitoring panels should reflect active hypoxia signaling, neovascularization, and vascular remodeling dynamics (10). A clinically successful stage-specific ncRNA panel should meet several criteria.

First, the panel should demonstrate robust discrimination in clinically meaningful adjacent-stage contrasts (e.g., moderate vs. severe NPDR, severe NPDR vs. early PDR), where real-world decision-making is most challenging (80). Second, its performance should remain stable after adjusting for major systemic confounders—particularly renal dysfunction and generalized inflammatory burden—which frequently co-exist in advanced diabetes and may distort circulating ncRNA profiles (82). Third, the panel should provide incremental value beyond imaging-based assessments, ideally demonstrated through external validation and interpretable performance metrics such as AUC improvement, calibration stability, and net reclassification indices (12). Finally, biological coherence should align with statistical performance; specifically, the selected ncRNAs should reflect coordinated regulatory pathways consistent with stage-specific retinal pathophysiology rather than being chosen based on arbitrary statistical criteria (76).

Although future advances may refine panel construction, current evidence is limited in cohort size and cross-validation strength. Therefore, the emphasis should remain on biologically grounded, stage-resolved network panels rather than prematurely moving toward highly individualized precision models.

## 9 Current limitations and future directions toward clinical translation

### 9.1 Current limitations and requirements for clinical applicability

Despite growing evidence supporting circulating ncRNAs as potential biomarkers for diabetic retinopathy (DR), several structural limitations currently hinder clinical translation. Most studies remain exploratory, with modest sample sizes and heterogeneous methodologies (12). The lack of reproducibility across cohorts and limited external validation restrict generalizability. Furthermore, many candidate markers are evaluated cross-sectionally, with insufficient evidence for stage-resolved predictive value—particularly at adjacent-stage transition points (e.g., moderate→severe NPDR or severe NPDR→early PDR) (80).

For clinical integration, stage-specific ncRNA panels should be positioned as decision-support tools that complement—rather than replace—imaging-based assessments. In screening settings, a blood-based panel could serve as a pre-imaging triage tool to prioritize high-risk patients for expedited fundus or OCT evaluation, or as a reflex test when imaging access is limited (75). In moderate NPDR, risk-stratification panels may inform surveillance intensity by identifying individuals likely to progress toward severe NPDR, thereby supporting personalized follow-up intervals and earlier referrals. At the boundary between severe NPDR and early PDR, progression-monitoring panels could be deployed longitudinally to detect imminent angiogenic transitions and to support timely escalation of care, provided that their incremental value over established imaging markers is prospectively validated (10). Clinical translation will therefore require not only statistical significance but also demonstrable workflow utility, stage-resolved discrimination, and added value beyond existing imaging-based risk models.

### 9.2 Insufficient stage-stratified cohorts and methodological gaps

A major barrier is the scarcity of rigorously stage-stratified cohorts. Many studies dichotomize patients into ‘DR’ versus ‘no DR’ or broadly classify NPDR and PDR without granularity across mild, moderate, and severe NPDR. This limitation hinders the evaluation of clinically decisive transition points (12, 80). Such practices restrict mechanistic insights into dynamic molecular transitions along the DR continuum and weaken the relevance of reported biomarker performance. Patient-level confounding represents an additional challenge for circulating ncRNA biomarker studies. Age, renal function, glycemic control, systemic inflammatory status, diabetes duration, and medication use may influence circulating ncRNA profiles independently of retinal pathology. Importantly, several of these factors also co-vary with DR severity; for example, patients with advanced DR may have longer diabetes duration, greater systemic comorbidity burden, or impaired renal function. Apparent stage-associated ncRNA differences may therefore partly reflect these correlated clinical characteristics rather than retinal disease severity alone. Future studies should report these variables systematically and assess whether candidate ncRNA associations remain robust after appropriate multivariable adjustment (6, 82).

Methodological heterogeneity further limits reproducibility. Extracellular vesicle (EV) research encounters technical variability, including inconsistent isolation protocols, differences in purity assessment, and a lack of harmonized reporting standards (78). In this context, standardization should be understood not just as procedural uniformity, but as reproducible analytical control across the pre-analytical, analytical, and post-analytical phases (summarized in **Box 2**). This includes hemolysis quality control to prevent contamination from erythrocyte-derived miRNAs, adherence to MISEV-aligned EV characterization criteria, transparent normalization strategies for circulating ncRNA quantification, and pre-specified staging definitions with clearly defined clinical endpoints (32, 81). Notably, among the primary circulating miRNA studies summarized in **Tables 4** and **1**, few explicitly reported hemolysis assessment or exclusion criteria, indicating that erythrocyte-derived contamination remains an undercontrolled confounder in the current literature.

Box 2 **Minimum standardization checklist for translational ncRNA biomarker studies.**

- Hemolysis quality control (e.g., spectrophotometric assessment or erythrocyte-miRNA markers)

- EV isolation with MISEV-aligned characterization (size distribution, marker profiling, purity metrics)
- Transparent and reproducible normalization strategy (spike-in controls, endogenous references, or global mean approaches justified *a priori*)
- Pre-specified clinical endpoints and detailed staging protocol (adjacent-stage contrasts defined before analysis)
- Reporting and adjustment for major clinical confounders (age, renal function, glycemic control, inflammatory status, diabetes duration, medication use, and relevant comorbidities)
- External validation/multi-center replication

Longitudinal datasets are also limited, restricting the ability to determine whether ncRNA alterations preceded structural progression or merely reflect established pathology (10). Without severity-stratified designs, standardized EV methodologies, and longitudinal validation, translational reliability remains constrained.

### 9.3 Future research directions: severity-stratified, longitudinal, and imaging-integrated validation

Future investigations should adopt severity-stratified study designs aligned with detailed clinical staging, incorporating pre-specified comparisons that prioritize contrasts between adjacent stages rather than merely comparing ‘DR vs non-DR’ (80). Longitudinal follow-up is essential to evaluate temporal dynamics and predictive performance, particularly for identifying molecular signals that precede angiogenic transitions (10). Integrating imaging endpoints—such as standardized fundus photography grading and OCT/OCTA-derived structural and ischemic measures—will enhance biological relevance and clinical interpretability (83). Validation should focus on the panel level, assessing whether multi-ncRNA networks demonstrate consistent stage-specific discrimination across independent cohorts and after adjustment for major clinical confounders (12). Such structured validation strategies will be critical for advancing from exploratory association studies to clinically actionable biomarker systems. Computational approaches, including machine learning-based feature selection (e.g., LASSO regression, random forest ranking) and multi-omics integration, may optimize the composition of multi-ncRNA panels and improve classification performance in high-dimensional datasets (84). However, these methods should serve as tools to refine biologically grounded panels rather than substitutes for mechanistic understanding. Stage classification frameworks must remain grounded in ncRNA regulatory biology—reflecting established pathogenic axes of inflammation, hypoxia, and angiogenesis—to ensure that statistically derived panels retain biological interpretability and clinical coherence (76).

## 10 Conclusions

### 10.1 Distinction from therapeutically oriented ncRNA reviews

Unlike many existing reviews that primarily focus on the therapeutic modulation of ncRNAs—such as miRNA inhibition, replacement strategies, or ncRNA-targeted interventions—this review adopts a biomarker-centered perspective (75). While therapeutic applications are biologically compelling, they are constrained by delivery barriers, off-target concerns, and the practical complexities of sustained intraocular or systemic modulation (9). In contrast, circulating ncRNAs provide a minimally invasive insight into retinal pathophysiology (10). Therefore, our emphasis is not on manipulating ncRNAs to alter disease progression, but on evaluating how stage-dependent ncRNA signatures can reflect, stratify, and monitor disease status across clinically meaningful severity boundaries. By shifting the focus from intervention to detection and monitoring, this review positions ncRNAs within a clinically grounded framework aligned with real-world decision points.

### 10.2 Stage-specific biomarker focus and the hierarchical role of miRNA, lncRNA, circRNA, and EVs

This review emphasizes the development of stage-resolved biomarkers rather than generalized DR-associated signatures. miRNAs currently represent the most mature ncRNA biomarker class, with the strongest evidence for stage-associated patterns and selected stage-discriminatory signals (12). In contrast, lncRNAs and circRNAs serve as complementary context layers that enhance the interpretability and robustness of miRNA-centered panels within ceRNA networks (76). Extracellular vesicles (EVs) further support translation by stabilizing ncRNA signals in biofluids and potentially enriching disease-relevant cargo (10). However, the association with EVs should primarily be interpreted as a means of stabilizing and potentially enriching disease-relevant signals, rather than as definitive proof of retinal specificity (79). Together, these elements define a hierarchical framework in which miRNAs provide core effector readouts, lncRNAs and circRNAs refine network context, and EV compartments improve analytical stability for circulating biomarker development.

### 10.3 Conceptual conclusion: toward stage-aware, network-informed panels

As argued throughout this review and outlined in the panel framework of Section 8, the path toward clinical translation requires stage-aware, network-informed biomarker panels rather than merely expanding individual candidate lists. Three unresolved gaps will determine whether this framework achieves clinical utility: (i) the identification and validation of ncRNA signals at adjacent-stage transition points—particularly the boundary between moderate and severe NPDR, where current primary evidence remains scarce (80); (ii) the standardization of pre-analytical and analytical workflows, including hemolysis control, EV characterization, and normalization, for which adoption rates in the existing literature remain low (32, 81); and (iii) longitudinal, imaging-integrated cohort studies capable of demonstrating predictive performance and providing incremental value beyond conventional staging (83). Until these gaps are addressed, ncRNA panels will remain promising but unproven. Closing them will determine whether circulating ncRNA signatures can become decision-support tools integrated into DR care pathways.
